# Effect of hypercholesterolemia alone or combined with hypertension on the degree of coronary artery stenosis in patients with coronary heart disease angina pectoris: A medical records based retrospective study protocol

**DOI:** 10.1097/MD.0000000000022225

**Published:** 2020-09-18

**Authors:** Xiaoxue Xue, Yijia Liu, Mingjie Yang, Shuo Wang, Mengnan Huang, Shuming Gao, Yilan Xu, Shan Gao, Lin Li, Chunquan Yu

**Affiliations:** a Graduate School, Tianjin University of Traditional Chinese Medicine; b Editorial Department, Tianjin University of Traditional Chinese Medicine, Tianjin, China.

**Keywords:** coronary heart disease angina pectoris, Gensini score, hypercholesterolemia, hypertension, retrospective study

## Abstract

**Background:**

Coronary heart disease (CHD) is the leading cause of death globally. Angina pectoris is closely associated with coronary artery insufficiency, which seriously affects the quality of life and work of patients. Hypercholesterolemia and hypertension (HTN) are risk factors for CHD angina pectoris. The correlation between hypercholesterolemia with or without HTN and the severity of coronary arteries has not been clarified.

**Objective:**

To explore the correlation between hypercholesterolemia and the degree of coronary artery stenosis (CAS) of CHD angina pectoris, and to further research the influence of HTN on total cholesterol level and CAS, so as to provide guidance for clinical prevention and treatment.

**Methods:**

A multicenter, retrospective clinical study was conducted in the medical records management system of 6 hospitals in Tianjin. Patients who were suffered from CHD angina pectoris and aged from 35 to 75 years old are involved. They hospitalized in the Department of Cardiology between September 1, 2014, and September 1, 2019, and underwent coronary angiography. We divide patients into 3 groups based on the total cholesterol level, the degree of CAS is evaluated by Gensini score, and further divide them into 6 subgroups based on with or without HTN. Collect and analyze the demographics, laboratory information, clinical outcome data, and coronary angiographic data of patients.

**Conclusion:**

Through clinical research data, the study will help to provide guidance for the prevention and treatment of CHD angina pectoris complicated with diseases and promote further research.

## 1 Introduction

Coronary heart disease (CHD) is a heart disease that is caused by myocardial ischemia, hypoxia, or necrosis because of coronary stenosis, or occlusion of coronary atherosclerosis.^[1]^ Angina pectoris is typical in clinical and closely associated with inadequate coronary blood supply; it can seriously affect the life and work of patients and families. Therefore, the prevention and treatment of angina pectoris should be taken into consideration.^[2–4]^ According to a report released in 2017 by the International Cooperation Study on the Global Burden of Disease, CHD is the leading cause of death in all diseases globally, with an estimated 110 million people suffered from CHD and 8.92 million deaths due to CHD, and the prevalence continues to rise.^[1,5]^ Evidence-based medicine and a large number of clinical epidemiological investigations show that hypercholesterolemia and hypertension (HTN) are risk factors for CHD angina pectoris.^[6]^ Hypercholesterolemia has resulted in 4 million deaths and a disability-adjusted life loss of up to 88.7 million people globally.^[7]^ Through multiple studies of risk analysis of CHD angina pectoris, HTN is generally regarded as an important risk factor for CHD angina pectoris.^[8]^ Combined with Gensini scoring system, Coronary angiography (CAG) makes a precise diagnosis of the number, location, scope, and vascular wall of coronary artery stenosis (CAS),^[9]^ CAG is regarded as the best way to diagnose CHD and the most powerful method of evaluating the degree of CAS.^[10]^

According to the report, some scholars have conducted combined studies of hypercholesterolemia and HTN. Still, no studies have reported the influence of HTN on the correlation between hypercholesterolemia and the degree of CAS. Therefore, we conduct a retrospective study to explore the relationship between hypercholesterolemia and CAS and whether its relationship is affected by HTN. Through clinical data, we will provide guidance for the prevention and treatment of CHD angina pectoris complicated with diseases and promote further research.

## 2 Method

### 2.1 Study design

We carried out a multicenter, retrospective clinical study. The collaborative network of CHD research medical units formed by the research team includes the first affiliated hospital of Tianjin University of traditional Chinese medicine (TCM), the second affiliated hospital of Tianjin University of TCM, Tianjin Nankai Hospital, the affiliated hospital of Tianjin Institute of TCM, Tianjin Chest Hospital and Tianjin Medical University General Hospital. The cases of 1000 patients meeting the diagnostic, inclusion and exclusion criteria were collected through the hospital medical record management system.

### 2.2 Qualification criteria

#### 2.2.1 Diagnostic criteria

(1) Diagnostic criteria for CHD angina pectoris: Based on the guidelines formulated by the American College of Cardiology and the American Heart Association (ACC/AHA) in the Guidelines for the Treatment of Chronic Stable Angina Pectoris (2007) and the Guidelines for the Management of Patients with Unstable Angina Pectoris and Non-ST-Elevation Myocardial Infarction (2011), and referring to the Guidelines for the Diagnosis and Treatment of Chronic Stable Angina Pectoris in China in 2007 and the Guidelines for the Diagnosis and Treatment of Unstable Angina Pectoris and Non-ST-Elevation Myocardial Infarction in China in 2007, and the Standard for the Consensus of Integrative Medicine for Integrative Medicine for the Treatment of CHD (2019).^[11]^

Diagnosis can be given if it meets any 1 or more of the following:

1. There was a clear history of old myocardial infarction.
2. Previous CAG or coronary CTA examination showed that at least 1 major branch of the coronary artery has a lumen diameter stenosis ≥ 50%.
3. Those with ST-segment depression or T-wave inversion on ECG after coronary revascularization.

CHD unstable angina pectoris includes the following subtypes:

1. Resting angina pectoris: it occurs at rest and usually lasts for more than 20 minutes.
2. Initial angina pectoris: it newly occurs within 1 month, which can be manifested as the spontaneous seizure coexists with the labor seizure, and the pain grade is above III.
3. Aggravated exertional angina pectoris: it has a previous history, with the worsening of symptoms, frequent attacks, prolonged duration, or lower pain threshold in the last 1 month.
4. Variant angina pectoris: it often happens spontaneously. It is characterized by transient ST-segment elevation, most of which relieve spontaneously, only a few develop into myocardial infarction.

(2) Diagnostic criteria of hypercholesterolemia:
  According to the Expert Consensus on Diagnosis and Treatment of Dyslipidemia with Integrated Traditional Chinese and Western Medicine,^[12]^ hypercholesterolemia is diagnosed when total cholesterol (TC) ≥ 5.18 mmol/L. Based on the TC level, we divide patients into 3 groups: patients with TC < 5.18 mmol/L belong to normal TC (NTC) group, 5.18 ≤ TC < 6.19 mmol/L is borderline-high TC (BHTC) group, those with TC ≥ 6.19 mmol/L belong to high TC (HTC) group.
(3) Diagnostic criteria of HTN:
  If the patients have been diagnosed with HTN or are currently taking antihypertensive drugs, we will classify they as HTN group.^[13]^ Otherwise, we will redefine HTN as systolic blood pressure (BP) ≥ 130 mm Hg or diastolic BP ≥ 80 mm Hg according to the recently published 2017 ACC/AHA guidelines.^[14]^

#### 2.2.2 Inclusion criteria

1. Patients aged from 35 to 75 years old, male or female.
2. Those who were hospitalized between September 1, 2014, and September 1, 2019.
3. Those with CHD angina pectoris and have undergone CAG in the inpatient department of internal cardiovascular medicine.
4. The diagnosis of hypercholesterolemia, CHD angina pectoris and HTN is accorded with the diagnostic criteria.

#### 2.2.3 Exclusion criteria

1. Those with other cardiac diseases, gastroesophageal reflux disease, or hiatal hernia, neurosis, spinal, or vertebral artery cervical spondylosis, hyperthyroidism, climacteric syndrome, etc.
2. Those with myocarditis, cardiomyopathy, acute myocardial infarction, third degree of heart failure, severe heart valve disease, major diseases such as malignant tumor and serious metabolic diseases, liver failure, or renal failure.
3. Those with psychotic disorders or cognitive dysfunction.
4. Those of childbearing age and have fertility requirements, pregnant women, or lactating women.
5. Those allergic to the iodine contrast medium.
6. Those who are not suitable for the study, as judged by the researcher.

### 2.3 Research group

**Figure 1:**
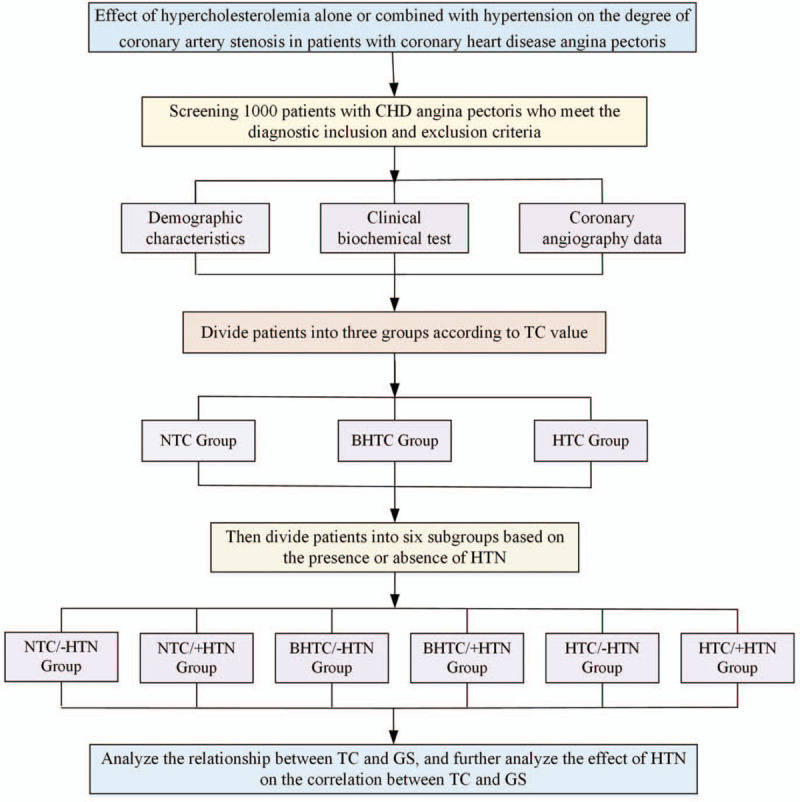
The flow diagram of effect of hypercholesterolemia alone or combined with hypertension on the degree of coronary artery stenosis in patients with coronary heart disease angina pectoris: a medical records based retrospective study.

Based on the TC level, we divide patients into 3 groups, and the correlation between the TC and the degree of CAS is evaluated. With or without HTN will be further stratified to assess the effect of with or without HTN on the correlation between TC levels and the degree of CAS. The 6 subgroups are: NTC/-HTN group, NTC/+HTN group, BHTC/-HTN group, BHTC/+HTN group, HTC/-HTN group, HTC/+HTN group. The NTC/-HTN group is taken as the reference group. The flow diagram is shown in Figure 1.

### 2.4 Observation indicators

In the hospital medical record management system, we collect the clinical data of the first day of hospitalization of the patients, which include demographics such as age, gender, ethnicity, weight, smoking, medical history, BP; clinical biochemical tests such as fasting plasma glucose, glycated hemoglobin, TC, Triglyceride, high density lipoprotein cholesterol, low density lipoprotein cholesterol, C-reactive protein, Fibrinogen and other indicators; CAG data in imaging examination.

According to the location and degree of the lumen stenosis, the score of the degree of each CAS is calculated. In order to calculate Gensini score (GS), the percentage diameter of the stenosis is assigned based on the severity of the stenosis (from 0–32): 1 point for stenosis ≤ 25%, 2 points for stenosis 26% to 50%, 4 points for stenosis 51% to 75%, 8 points for stenosis 76% to 90%, 16 points for stenosis 91% to 99%, and 32 points for stenosis 100%. Lesion site score: 5 points for left main coronary artery, 2.5 points for proximal left anterior descending (LAD), 1.5 points for middle LAD, 1.0 point for distal LAD, 1.0 point for the first diagonal branch, 0.5 points for the second diagonal branch, 2.5 points for proximal left circumflex (LCX), 1.0 point for distal LCX, 1.0 point for blunt LCX, 1.0 point for posterior descending branch, 0.5 points for posterior branch and 1.0 point for right coronary artery. Multiply the stenosis score by the lesion site score, then add all the scores we will get the final GS, which is defined by 2 independent doctors.^[15,16]^ The data of CAG were separately defined by more than 2 interventional doctors who did not know the rest data of patients, and a third doctor will participate in the analysis if necessary. The GS rule is shown in Table 1.

**Table 1:**
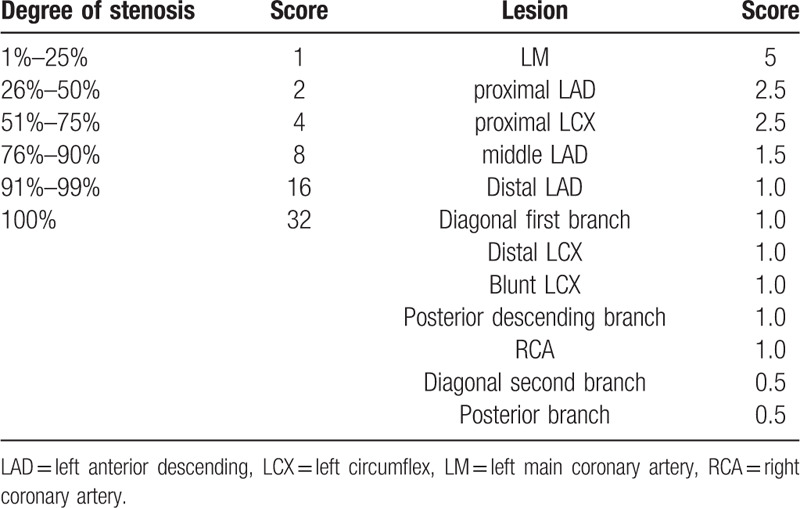
The Gensini score rule.

### 2.5 Data management

#### 2.5.1 Quality assurance

To carry out the research smoothly, the research team will hold a special clinical training meeting before the formal launch of the clinical trial and provide researchers with centralized training in each center.

Conduct training on the project implementation program and standard operation procedure (SOP) to make every researcher has an intimate knowledge of the process of study and specific operation method, so as to promote the consistency of internal observations among researchers and between observers, and ensure the reliability of clinical research conclusions.

Sign the investigator's statement and establish SOP for observation and quality control of various indicators in the laboratories of participating hospitals.

Quality control measures of each center. Conduct regular audits of the centers to control the bias between the centers.

#### 2.5.2 Original data management

We use the paper case report form (CRF); those will be valid after being checked and approved by the researchers. All CRFs, as well as the modification process, will be preserved.

#### 2.5.3 Data entry

Double data entry is performed using Epidata 3.1 (Jens M. Lauritsen, Odense, Denmark) database, and 2 qualified professionals will enter the data into the computer separately. Before data entry, we will conduct specialized entry training for relevant individuals. They will enter data based on the entry guide formulated according to the research content.

#### 2.5.4 Medical coding

Medical coding is performed by specialized data managers with a medical background. The coding content is medical history. The medical history will be coded according to MedDRA dictionary (Version 13.0). The encoded documents will be sent to the main researchers for review and confirmation in the form of electronic documents.

#### 2.5.5 Writing of data verification and answers to questions

We will carefully check the entered data one by one based on the data verification plan (SAP). SAP should be signed and approved by the data managers and statisticians.

We use 2 verification methods, including systematic verification and manual medical verification. The former mainly uses SAS 9.3 (SAS Institute Inc., Cary, NC) software to write programs and then generates a data clarification form (DCF). All data queries discovered by the 2 methods will be entered into the data query database, a formal DCF will be produced by the data management. DCF will be handed over to research inspectors after being checked. After re-inspection, inspectors will submit DCF to researchers in each center for further inspection and reply. Only after receiving the DCF with the researcher's signature and date, can we modify the database. It will be placed in the project-special file cabinet for storage. All modifications to the database will be recorded.

#### 2.5.6 Database locking

The database will be locked when it meets the following requirements:

All questions (including those raised at the data review meeting) have been resolved, and the database has been updated.
No new questions were found in the data verification.
The medical coding has been completed and approved by the applicant.
The crowd division plan has been approved.
SAP has been finalized and approved by the project leader.

The head of DSMB, statistician and data administrator jointly sign the database locking form, while the database is locked. After the database is locked, any change of data must be approved by the project leader and filed by the data management unit.

**Figure 2:**
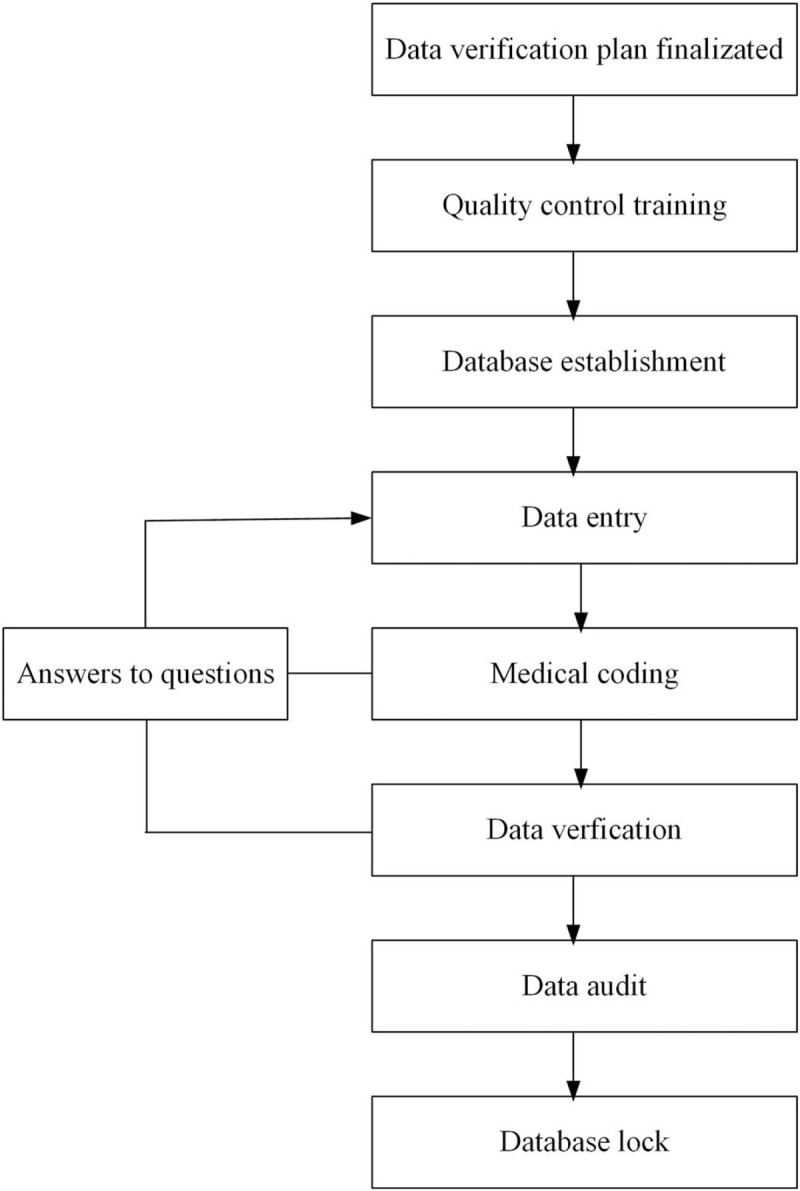
The overall flow of data management.

The overall process of data management is shown in Figure 2.

### 2.6 Statistical analysis

Before performing statistical analysis, we shall invite statistical analysts to make plans and specify detailed steps, then carefully examine the data collected. All statistical analysis processes are programmed and archived for future reference.

#### 2.6.1 Statistical methods

1. Continuous variables conforming to the normal distribution are expressed by standard deviation and mean, and the difference between groups is analyzed by one-way ANOVA.
2. If it conforms to non-normal distribution, we use the median, upper quartile (Q1), lower quartile (Q3), 95% confidence interval to express, and rank-sum test (Wilcoxon method) for analysis.
3. The classified data are expressed in terms of quantity and percentage and analyzed by the χ^2^ test and Fisher exact probability method.
4. If considering the influence of baseline confounding factors, logistic regression analysis is used. The correlation between variables is analyzed by Spearman or Pearson correlation.

We use SPSS version 23.0 (IBM Corporation, Armonk, NY) to conduct all analyses. The general statistical test uses a bilateral test, and bilateral *P* < .05 is considered statistically significant.

### 2.7 Morality and communication

The research of this subject must be approved by the ethics committee of each central research unit before the clinical research begin. The research plan was approved by the Ethics Committee of Tianjin University of TCM (TJUTCM-EC20200005). Informed request for consent was abandoned. It has been registered in the China Clinical Trial Registry with the registration number: ChiCTR2000033863. The patient's personal information will be protected and all data will be collected anonymously. The findings of the study will be published in peer-reviewed journals and disseminated electronically and printed.

## 3 Discussion

In China, the death rate of cardiovascular disease still ranks first, accounting for more than 40% of the death rate of residents, and it is much higher than the death rate of tumors and other diseases.^[17]^ According to a report released by the Chinese Expert Consensus on the Comprehensive Management of BP and lipids in hypertensive patients (2019), there are currently about 245 million HTN patients in China. Among the patients with HTN screened by the population, those with at least 1 dyslipidemia accounted for 41.3%, and those with hypercholesterolemia were 9.9%.^[18,19]^

Studies have found that hypercholesterolemia and HTN have both separate and synergistic effects on the occurrence of CHD. The combination of the 2 effects is not a simple superposition effect, but an amplification effect or a product effect. The synergy may be realized through several direct and indirect mechanisms. Endothelial injury theory or oxidized LDL endothelial injury theory may be the initiating mechanism of cholesterol deposition on the arterial wall. Recent studies have found that endothelial cells can maintain hemodynamic stability and affect the process of CHD by producing nitric oxide (NO) and angiotensin-II (AT-II).^[20]^

This study has the following advantages:

(1) The design idea is novel. A study^[13]^ has discussed the effects of diabetes mellitus alone or combined with HTN on CAS. Still, there are few reports focus on the correlation between HTN and hypercholesterolemia and GS of CAS.
(2) Standardized diagnostic, inclusion and exclusion criteria. This research protocol sets strict inclusion and exclusion standards and adopts the latest diagnostic standards at home and abroad. For example, compared with the diagnostic standard for HTN in the JNC 7 report,^[21]^ this research uses the latest diagnostic standard for HTN concerning the recently published 2017 ACC/AHA guidelines.
(3) The sources of clinical data for this study are extensive. The data of this study comes from multi-centers, and the results are more representative.
(4) The design method is accurate. Before performing statistical analysis, we shall invite statistical analysts to make plans and carefully examine the collected clinical data.
(5) Strict quality control. Before collecting clinical data, researchers should be trained in a unified way. All CRFs will not take effect until they have been reviewed and signed by researchers in a unified way. Data entry will be done by trained professionals in pairs for the accuracy of the data entry, and the objectivity of the results.

Though we have set strict rules on possible problems of the study program, because of the complexity and variability of the clinical study, some research results may still be affected, and we will further improve the protocol based on actual research. The study will provide guidance for the prevention and treatment of CHD angina pectoris complicated with the disease through clinical research data, and promote its further research.

## Author contributions

**Data curation:** Chunquan Yu, Shan Gao, Lin Li.

**Investigation:** Xiaoxue Xue, Yijia Liu, Lin Li.

**Methodology:** Chunquan Yu.

**Resources:** Mengnan Huang.

**Software:** Shuo Wang, Shuming Gao.

**Statistics:** Shuo Wang, Shuming Gao.

**Writing – original draft:** Xiaoxue Xue, Yijia Liu, Mingjie Yang.

**Writing – review & editing:** Chunquan Yu, Yilan Xu.

## Abbreviations

Abbreviations: ACC/AHA = American College of Cardiology and the American Heart Association, BHTC = borderline-high TC, BP = blood pressure, CAG = coronary angiography, CAS = coronary artery stenosis, CHD = coronary heart disease, CRF = case report form, DCF = data clarification form, GS = Gensini score, HTC = high TC, HTN = hypertension, LAD = left anterior descending, LCX = left circumflex, NTC = normal TC, SAP = data verification plan, TC = total cholesterol, TCM = traditional Chinese Medicine.
